# ADVANCING LATE-LIFE TRAUMA-INFORMED CARE EDUCATION: DEVELOPMENT AND EVALUATION OF AN EDUCATIONAL PODCAST

**DOI:** 10.1093/geroni/igac059.1982

**Published:** 2022-12-20

**Authors:** Rachel Weiskittle, Lola Baird, Hannah Bashian, Anica Pless Kaiser, Kelly O'Malley, Anna Etchin, Jennifer Moye

**Affiliations:** University of Colorado Colorado Springs, Colorado Springs, Colorado, United States; Boston VA Medical Center, Jamaica Plain, Massachusetts, United States; VA Boston HCS, Boston, Massachusetts, United States; VA Boston Medical Center, Jamaica Plain, Massachusetts, United States; VA Boston Healthcare System, Boston, Massachusetts, United States; VA Boston Medical Center, Jamaica Plain, Massachusetts, United States; VA Boston Healthcare System, Boston, Massachusetts, United States

## Abstract

Posttraumatic stress disorder (PTSD) may emerge or reemerge in the context of life-threatening illness, retirement, and life review, leading to complications in disease management and end-of-life care. Symptoms of PTSD in later life can be misattributed or overlooked by healthcare professionals, caregivers, and the older adults experiencing PTSD themselves. The “Talking Later” podcast was developed as an accessible educational product to improve recognition and trauma-informed responses to late-life PTSD. Each of the ten episodes had distinct goals and content objectives, which were identified through problem identification and needs assessment by a multidisciplinary team of geriatric clinicians. In the first three months of its publication in November 2021, the podcast has been downloaded by 1,473 unique listeners across 19 countries. The podcast was evaluated via feedback survey (N=39). Approximately 97% of respondents reported the episodes as engaging and informational. 87% stated that no more than general knowledge of PTSD was required to enjoy the podcast. Qualitative analysis of open-ended feedback items found that participants were interested in learning about additional comorbidities and diversity issues related to late-life trauma reengagement. The present poster will additionally describe ongoing efforts to address these and other feedback responses with the ongoing development of a second season of the podcast.

